# Biomarkers of endothelial dysfunction predict sepsis mortality in young infants: a matched case-control study

**DOI:** 10.1186/s12887-018-1087-x

**Published:** 2018-03-23

**Authors:** Julie Korol Wright, Kyla Hayford, Vanessa Tran, Gulam Muhammed Al Kibria, Abdullah Baqui, Ali Manajjir, Arif Mahmud, Nazma Begum, Mashuk Siddiquee, Kevin C. Kain, Azadeh Farzin

**Affiliations:** 1 Tropical Disease Unit, Division of Infectious Diseases, Department of Medicine, University of Toronto, Toronto, ON Canada; 2 Sandra Rotman Centre for Global Health, University Health Network-Toronto General Hospital, Department of Medicine, University of Toronto, Toronto, ON Canada; 3 Department of International Health, Bloomberg School of Public Health, Johns Hopkins University, Baltimore, MD USA; 4 International Centre for Maternal and Newborn Health, Department of International Health, Bloomberg School of Public Health, Johns Hopkins University, Baltimore, MD USA; 5 Department of Pediatrics, Sylhet MAG Osmani Medical College Hospital, Sylhet, Bangladesh; 6 Dhaka Shishu (Children’s) Hospital, Sher-E-Bangla Nagar, Dhaka, Bangladesh; 7 Division of Neonatology, Department of Pediatrics, Johns Hopkins University School of Medicine, Baltimore, MD USA

**Keywords:** Neonatal Sepsis, Endothelial activation, Angiopoietins, Biomarkers

## Abstract

**Background:**

Reducing death due to neonatal sepsis is a global health priority, however there are limited tools to facilitate early recognition and treatment. We hypothesized that measuring circulating biomarkers of endothelial function and integrity (i.e. Angiopoietin-Tie2 axis) would identify young infants with sepsis and predict their clinical outcome.

**Methods:**

We conducted a matched case-control (1:3) study of 98 young infants aged 0–59 days of life presenting to a referral hospital in Bangladesh with suspected sepsis. Plasma levels of Ang-1, Ang-2, sICAM-1, and sVCAM-1 concentrations were measured at admission. The primary outcome was mortality (*n* = 18); the secondary outcome was bacteremia (*n* = 10).

**Results:**

Ang-2 concentrations at presentation were higher among infants who subsequently died of sepsis compared to survivors (aOR 2.50, *p* = 0.024). Compared to surviving control infants, the Ang-2:Ang-1 ratio was higher among infants who died (aOR 2.29, *p* = 0.016) and in infants with bacteremia (aOR 5.72, *p* = 0.041), and there was an increased odds of death across Ang-2:Ang-1 ratio tertiles (aOR 4.82, *p* = 0.013).

**Conclusions:**

This study provides new evidence linking the Angiopoietin-Tie2 pathway with mortality and bacteremia in young infants with suspected sepsis. If validated in additional studies, markers of the angiopoietin-Tie2 axis may have clinical utility in risk stratification of infants with suspected sepsis.

**Electronic supplementary material:**

The online version of this article (10.1186/s12887-018-1087-x) contains supplementary material, which is available to authorized users.

## Background

Globally, sepsis and its sequelae are leading causes of childhood morbidity and mortality. Among neonates, sepsis is a major contributor to an estimated 2.6 million annual deaths and accounts for approximately 3 % of all disability adjusted life years [1, 2]. Early recognition and initiation of antimicrobial therapy are essential to reduce the morbidity and mortality of neonatal sepsis. However, early signs of sepsis are subtle and we currently lack diagnostic tools to enable rapid triage and management of at-risk infants, especially in low-resource settings where 99% of the world’s neonatal deaths occur [3, 4].

Septic shock represents a final common pathway for a variety of life-threatening infections and culminates in multiple organ failure and death. While the pathobiology of septic shock is complex and incompletely understood, dysregulated systemic inflammatory responses and endothelial dysfunction are believed to play key roles [5–7]. These altered host responses are associated with decreased systemic vascular resistance, loss of endothelial integrity, and microvascular leak, which compromise tissue perfusion and organ function [8].

The Angiopoietin proteins are a family of endothelium-derived angiogenic factors that have potent effects on the vascular endothelium. Angiopoietins interact with their cognate tyrosine kinase receptor, Tie2, expressed on the luminal endothelium. When bound by Angiopoietin-1 (Ang-1), Tie2 signaling promotes endothelial quiescence by enhancing cell survival, maintaining stable adherens junctions through the inhibition of nuclear factor kappa-light-chain-enhancer of activated B cells (NFkB), and downregulating pro-inflammatory cell adhesion molecules including intercellular adhesion molecule-1 (ICAM-1) and vascular cell adhesion molecule-1 (VCAM-1) [9–11]. Endothelial injury stemming from a range of insults including inflammation and hypoxia, stimulates exocytosis of endothelial Weibel-Palade bodies and the release of Angiopoietin-2 (Ang-2) [9, 12]. Ang-2 generally functions as a competitive antagonist for Ang-1 binding to Tie2. Under the influence of Ang-2, activated endothelial cells increase the surface expression of cellular adhesion molecules including ICAM-1 and VCAM-1, and undergo alterations in endothelial cell-cell junctions resulting in microvascular leak [9, 13, 14].

During embryonic, fetal, and early postnatal development, the Angiopoietin-Tie2 axis regulates angiogenesis by directing blood vessel formation, remodeling, and stabilization [15] (reviewed in [16]). Beyond this developmental window the angiopoietin family of ligands continues to regulate important endothelial phenotypes. Multiple disease states are characterized by endothelial activation and microvascular leak including septic shock [17], the hemolytic uremic syndrome [18], toxic shock syndrome [19], malaria [20–24], dengue [25], and acute lung injury / acute respiratory distress syndrome [26–28]. Each of these life-threatening conditions also manifest alterations in Ang-2:Ang-1 plasma concentrations favoring Ang-2 antagonism of Tie2 signaling (reviewed in [29, 30]).

A growing body of evidence has delineated the role and temporal kinetics of Angiopoietin-Tie2 related endothelial activation in septic shock, multiorgan dysfunction, and death (reviewed in [29, 31]). Circulating levels of soluble ICAM-1 (sICAM-1) have been associated with mortality in ICU patients [32, 33], adult systemic inflammatory response syndrome (SIRS) and sepsis severity [33–35], and bacteremia [36]. However among neonates, this association is less consistent with some studies reporting no association between sICAM-1 and sepsis [37, 38], while others demonstrate a positive association [39–42], even in the early stages of sepsis [43]. Soluble-VCAM-1 (sVCAM-1) has been shown in some adult studies to be associated with sepsis [32, 34], whereas in neonates circulating sVCAM-1 was not associated with sepsis but rather only with bacteremia [43]. Taken together, these studies suggest that the Angiopoietin-Tie2 axis may have pleiotropic effects within the immature vascular endothelium of the neonate.

The Tie2 ligands, Ang-1 and Ang-2, have been studied for potential diagnostic and prognostic utility in sepsis. Among adult patients with severe sepsis admitted to the ICU, survivors had higher circulating Ang-1 levels and lower Ang-2 levels than non-survivors [7]. When plasma angiopoietin levels were assessed in adult patients with sepsis on presentation to the Emergency Department, admission Ang-2 levels were predictive of sepsis severity including septic shock and death [17]. Similar findings are documented in the pediatric literature, where Ang-2 levels were associated with sepsis and correlate with disease severity [28, 44–46]. However, none of these studies included neonates or young infants. Globally, and especially in resource-poor settings, children under two months of age bear a high burden of sepsis-related morbidity and mortality [47].

In this matched case-control study conducted at a pediatric referral facility in Bangladesh, young infants under the age of two months who were admitted to hospital with presumed sepsis were enrolled and circulating levels of Ang-2, Ang-1, sICAM-1, and sVCAM-1 were assessed from admission blood samples. We hypothesized that elevated levels of circulating Ang-2 at admission would correlate with clinical outcomes. The primary outcome was mortality and the secondary outcome was bacteremia. These angiogenic biomarkers were selected for study based on their mechanistic role in the pathophysiology of sepsis, and their potential to be predictive of outcome in this vulnerable population.

## Methods

### Study population

This matched case-control study was nested in an observational cohort study investigating the prognostic potential of circulating angiogenic and inflammatory biomarkers for identifying young infants at triage who are at risk of severe sepsis and death. The study was conducted at the Sylhet MAG Osmani Medical College Hospital (SOMCH), in Sylhet, Bangladesh between July 16, 2013 and December 31, 2014.

### Enrollment criteria

Children aged 0–59 days of life with suspected sepsis were recruited upon presentation to the SOMCH Pediatrics ward. Clinical suspicion of sepsis was based upon the assessment of the treating physician, and the patients’ parents/guardians were approached for study enrollment upon meeting the inclusion criteria. Inclusion criteria were based on the World Health Organization (WHO) Integrated Management of Childhood Illness (IMCI) algorithm [48] and included: 1) history of difficulty feeding, 2) history of convulsions, 3) movement only when stimulated, 4) respiratory rate of 60 breaths per minute or more, 5) severe chest indrawing, 6) temperature greater than 37.5 °C or less than 35.5 °C.

Infants were excluded if there was suspicion of a congenital disorder involving a major organ system, any suspected chromosomal abnormalities, or if their presentation was attributed to an acquired structural illnesses (eg. pneumothorax or necrotizing enterocolitis), intrapartum-related complications, or morbidities of prematurity and low birthweight. Infants were also excluded if no research specimen was collected or if there was inadequate follow up of the infant. Due to a low rate of positive blood cultures in enrolled infants, there was an interim amendment to the study protocol to exclude infants with antibiotic exposure within 24 h of presentation.

### Clinical management

All infants received standard clinical care during the study and were visited daily by the study physician while inpatients. Families who left against the treating physician’s recommendation and prior to clinical improvement were contacted after discharge using the provided mobile phone number. In cases where the family left prior to improvement and could not be reached in person or via phone follow up, the infants were categorized as insufficient follow up and excluded, as described above. Standard clinical care included supportive measures such as intravenous fluids, oxygen administration in cases of cyanosis, gavage enteral feeds, thermal support using infant incubators, and the provision of empiric antibiotics on clinical suspicion of sepsis. Common antibiotic regimens for the management of presumed sepsis included intravenous ampicillin and gentamicin, ampicillin and cefotaxime, or ceftazidime and amikacin. All infants enrolled in the study had blood cultures performed.

### Blood sample collection and processing for biomarker analysis and blood culture

Upon enrollment into the study, venipuncture was performed for collection of the research specimen and blood culture, which was provided at no cost for all enrolled participants. SOMCH is a tertiary care centre for a population with significant resource limitations and therefore many children presented with severe illness at the time of diagnosis. For ethical reasons, we ensured that specimen collection for blood culture and research purposes did not delay administration of the first dose of antibiotics. The timing of blood collection in relation to antibiotic administration was recorded as part of research data collection.

For blood cultures, 2.0 mL of venous blood was collected into Lysis-Direct Plating (LDP) tubes on admission. Alternatively, in 29 cases where LDP tubes were not available, eight samples were collected into BACTEC bottles, and 21 blood samples were inoculated into Tryptic Soy Broth for incubation. The protocol for the isolation and detection of bacteria was adapted from Saha et al. [49].

For the research specimen, 1–2 mL of venous blood was collected into an EDTA blood collection tube and transferred to the laboratory within two hours in a 4 °C container. Specimens were centrifuged for 10 min at 2500 rpm to separate plasma, which was collected into sterile cryovials and stored at -20 °C prior to being batch transferred in liquid nitrogen to the central laboratory in Dhaka for storage at -70 °C. The frozen plasma samples were transferred to Toronto, Canada for analysis.

### Biomarker testing

Plasma concentrations of Ang-1, Ang-2, sICAM-1, and sVCAM-1 were measured in duplicate by Enzyme Linked Immunosorbent Assay (ELISA) (DuoSets, R&D Systems, Minneapolis, MN) as described in [22]. Laboratory technicians were blinded to patient outcome. Sample dilutions were optimized for the detection of each protein using a dilution curve obtained using a selection of case and control plasma samples. The final ELISA plates were read by spectrophotometry at 405 nm with a correction of 570 nm. Concentrations were extrapolated from a 4-parameter non-linear regression curve using Gen5 software (v1.02.8). The range of detection for each biomarker was as follows: Ang-1 (1.562–100 ng/mL), Ang-2 (1.875–120 ng/mL), sICAM-1 (62.50–4000 ng/mL), sVCAM-1 (312.5–20,000 ng/mL). Results below the lower limit of detection were adjusted according to the formula: 1/2 * lower limit of detection for the biomarker in the diluted sample. Results above the upper limit of detection were assigned the value of the upper limit of detection in the diluted sample.

### Outcome definitions

The primary outcome of this study was death during the index admission; infants who were discharged home in anticipation of an imminent death were also included in this primary outcome. The primary controls, termed ‘Survivors’, were young infants in the study cohort without bacteremia who were observed for at least 48 h at the hospital with evidence of clinical improvement prior to discharge, or confirmation of improvement provided by the family after discharge. Controls were retrospectively selected at a 3:1 ratio matched on birth weight (±500 g) and age at admission by category (0–2, 3–6, 7–13, 14–27, or > 27 days of life) as these were potentially confounding variables.

The secondary outcomes for this study were 1) culture-confirmed bacteremia, and 2) a combined outcome of death or bacteremia. Controls for secondary outcomes were matched using the same criteria as the primary analysis and termed ‘Non-Bacteremia’ and ‘Controls’, respectively.

### Data analysis

Demographic characteristics, location and type of delivery, antibiotic exposure and clinical findings at admission were compared for those infants with the primary or secondary outcomes and their controls using bivariate conditional logistic regression for continuous variables and exact McNemar’s test for binary variables to account for matching. No informative missingness was observed for independent variables. Two missing values for temperature and lethargy were randomly imputed and sensitivity analyses were conducted. Non-normally distributed continuous variables were natural log transformed. Multivariate conditional logistic regression models were generated to estimate the association of log-transformed biomarker levels at admission for the primary outcome and secondary outcomes. Because there are no clinically informative cutoffs among young infants for these biomarkers, biomarker distribution were divided into tertiles and analysed for an association with the Death outcome. Adjusted odds ratios (aOR) and 95% confidence intervals (CI) are reported. Final model selection was based on variables selected a priori (sex) and variables that balance parsimony with model fit. There were no changes in inferences in the sensitivity analyses. Analyses were performed in Stata 14 (Stata Corporation, College Station, TX).

## Results

### Patient characteristics

Four hundred and twenty three infants admitted with sepsis met eligibility criteria for the parent study and of these, parental/guardian consent for enrollment was given for 420. Mortality among this cohort was 10.4% (44/420) and the rate of culture-confirmed bacteremia was 3.1% (13/420). Of the mortality cases, 9.1% (4/44) had culture-confirmed bacteremia.

**Fig. 1:**
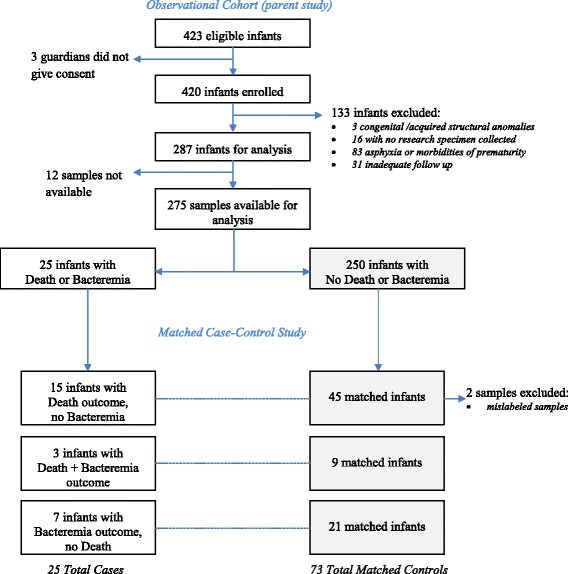
Study Flow Diagram. Infants in the matched case-control analysis included all infants from the Observational Cohort Study with an outcome of death (*n* = 18) or culture-confirmed bacteremia (*n* = 10) plus control infants who were randomly selected at a 3:1 ratio after matching on birthweight and age at admission

In total, angiogenic biomarkers were assessed in 98 infant plasma samples from the parent study cohort using a matched case-control design (Fig. 1). There were 18 primary outcomes (death) and 10 infants with culture-confirmed bacteremia, of which three died. Thus 25 infants fit the Combined Outcome group (death or bacteremia). Controls were selected from among the infants without bacteremia who survived to discharge at a 3:1 ratio for each outcome: 52 controls for the primary outcome, termed ‘Survivors’ (two samples were initially misclassified and excluded); and 30 controls for the Bacteremia group, termed ‘non-Bacteremia’. For the Combined Outcome group, there were a total of 73 combined controls, termed ‘Controls’.

The median age at admission was 14.5 days of life [inter-quartile range (IQR): 7, 27], and the median admission weight was 2.5 kg [IQR: 2.2, 3.0]. Males comprised 62% of the study population. All outcome groups had a significantly higher proportion of males compared with their respective controls (Table 1). Thirty-two per cent of infants had been born in a hospital and 10% had been delivered by Caesarean section. Between the Death and Survivor groups there were no statistically significant differences in these delivery characteristics; however, the rate of Caesarean section was significantly lower among the Bacteremia group and the Combined Outcome group compared to their controls (*p* = 0.039 and *p* = 0.014, respectively Table 1).

**Table 1:** Demographic and Clinical Characteristics of Enrolled Infants

	Deaths (n = 18)	Survivors (*n* = 52)	*p-value* ^*a,b*^	Bacteremia (n = 10)	Non-Bacteremia (*n* = 30)	*p-value* ^*a,b*^	Combined Outcome (*n* = 25)	Control (*n* = 73)	*p-value* ^*a,b*^
Demographic characteristics									
Age, median in days [IQR]	16.5 [9, 24]	17 [10, 27]	*0.552*	11 [4, 24]	10.5 [3, 16]	*0.951*	16 [8, 24]	14 [7, 27]	*0.482*
Weight, median in kg [IQR]	2.1 [1.9, 3.0]	2.4 [2.0, 2.8]	*0.059*	2.7 [2.5, 3.1]	2.7 [2.3, 3.0]	*0.455*	2.5 [2.0, 3.0]	2.5 [2.2, 3.0]	*0.294*
Male, % (#)	**67 (12)**	**58 (30)**	**< 0.001**	**90 (9)**	**55 (16)**	**< 0.001**	**72 (18)**	**59 (43)**	**< 0.001**
Birth characteristics									
Born in hospital, % (#)	22(4)	31 (16)	0.715	20 (2)	37 (11)	0.648	24 (6)	34 (25)	0.451
Delivery by Caesarian section, % (#)	6 (1)	16 (9)	0.169	**0 (0)**	**7 (2)**	**0.039**	**4 (1)**	**13 (9)**	**0.014**
Clinical findings at admission									
Temperature, ° C [IQR]	36.7 [36.2, 37.8]	37.6 [36.7, 37.8]	0.062	37.5 [37.1, 38.6]	37.7 [37.3, 38.2]	0.435	37.2 [36.4, 37.8]	37.7 [36.9, 38.1]	0.147
Lethargy, % (#)	38.9 (7)	18.0 (9)	0.824	20.0 (2)	13.3 (4)	0.388	32 (8)	15 (11)	0.345
Respiratory rate, breaths per minute [IQR]	66 [42,75]	57 [48.5, 69.5]	0.729	64.5 [56, 78]	62 [53, 66]	0.476	66 [52, 75]	61 [49, 68]	0.481
Antibiotics prior to blood culture, % (#)	50 (9)	38 (20)	0.215	40 (4)	50 (15)	0.078	**40 (10)**	**45 (33)**	**0.013**

*Ang-1* angiopoietin-1, *Ang-2* angiopoietin-2, *Ang2:1* ratio of Ang-2 to Ang-1, *sICAM* soluble intercellular adhesion molecule-1, *sVCAM* soluble vascular adhesion molecule-1, *IQR* inter-quartile range

^a^Test for differences between groups not reported for variables that were used to match cases and controls (age and weight)

^b^Continuous variables compared using bivariate conditional logistic regression. Binary variables compared using exact McNemar’s test

Comparisons with *p*-values less than or equal to 0.05 marked in bold

There were no significant differences in baseline clinical parameters at admission (temperature, respiratory rate, or lethargy) between any of the outcome groups and their respective controls (Table 1). The median time from admission to death was 19.5 h [IQR: 9, 40 h]. Forty-four per cent of infants in this study cohort received antibiotics within seven days prior to venipuncture for blood culture sample collection. Overall, prior antibiotic exposure was significantly associated with the Control group (*p* = 0.013) (Table 1). There was no statistically significant difference in the probability of positive culture result based on blood culture method in both the unmatched parent-study cohort (*n* = 420) and the matched case-control study population (*n* = 98). Among the 10 bacterial isolates from blood cultures, seven were gram positive organisms and three were gram negative (Additional file 1: Table S1). Due to the variety of organisms and low overall rate of bacteremia, correlations between pathogens and mortality or biomarker levels were not conducted.

### Increased concentrations of circulating Ang-2, Ang-2:Ang-1 ratio, and sICAM-1 at admission are associated with infant mortality

**Fig. 2:**
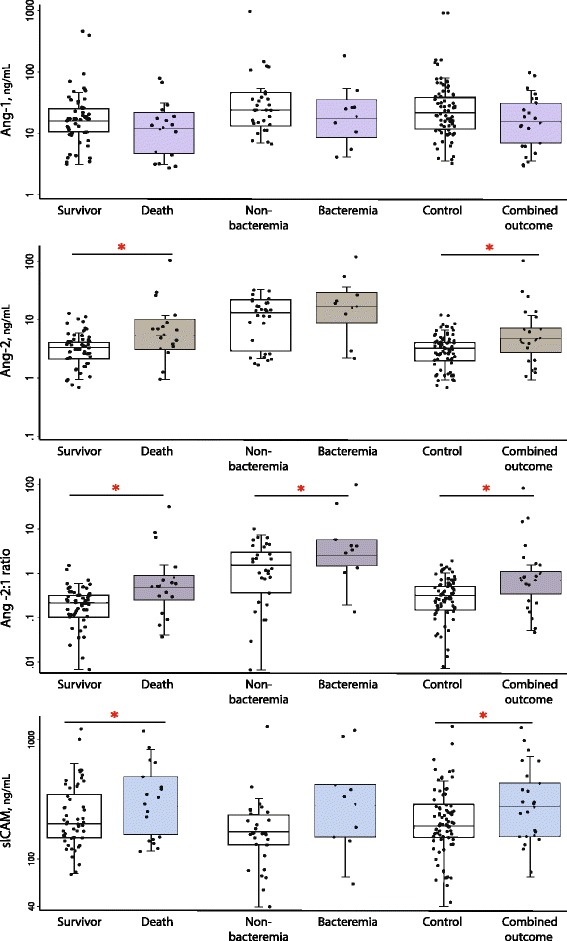
Distribution of angiogenic biomarkers by mortality, bacteremia, and combined outcomes. Circulating levels of Ang-2, sICAM-1 and the Ang-2:1 ratio at admission were associated with increased risk of death and the combined outcome of death and bacteremia. Only the Ang-2:1 ratio was significantly associated with bacteremia. Ang-1 levels at admission were not associated with any of the clinical outcomes. * indicates *p* < 0.05 based on conditional logistic regression adjusting for relevant confounding variables: sex and lethargy for mortality outcome, sex for bacteremia outcome, and sex, lethargy and temperature for combined case outcome

Median plasma Ang-2 concentration at presentation was significantly higher among infants with suspected sepsis who subsequently died compared to those who survived (5.4 ng/mL [IQR: 3.1, 10.1] vs 3.3 ng/mL [IQR: 2.1, 4.1], aOR 2.50, *p* = 0.024) (Fig. 2; Table 2). The relative odds of death increased with each tertile increase of plasma Ang-2 concentration, and the trend approached statistical significance (p-trend = 0.061) (Table 3). There was no statistically significant difference in median plasma Ang-1 levels among infants who died versus those who survived (11.7 ng/mL [IQR: 4.7, 21.5] vs 15.8 ng/mL [IQR: 10.5, 25.0], aOR 0.51, *p* = 0.119) (Fig. 2; Table 2). All models with the primary outcome were adjusted for prior antibiotic exposure, lethargy, and sex.

**Table 2:** Median values of angiogenic biomarkers and odds of death or bacteremia

Biomarker	Death (n = 18) median [IQR]	Survivor (n = 52) median [IQR]	aOR (95% CI)	p -value*	Bacteremia (n = 10) median [IQR]	Non-bacteremia (n = 30) median [IQR]	aOR (95% CI)	p -value*	Combined Outcome (n = 25) median [IQR]	Control (n = 73) median [IQR]	aOR (95% CI)	p -value*
Ang-1 (ng/mL)	11.7 [4.7, 21.5]	15.8 [10.5, 25.0]	0.51 (0.22, 1.19)	0.119	11.2 [6.0, 20.4]	14.7 [8.7, 26.2]	0.49 (0.17, 1.44)	0.194	11.2 [*5.7, 20.4*]	*15.2 [8.9, 25.0]*	*0.54 (0.26, 1.10)*	*0.089*
Ang-2 (ng/mL)	**5.4 [3.1, 10.1]**	**3.3 [2.1, 4.1]**	**2.50 (1.13, 5.54)**	**0.024**	*4.1 [2.6, 6.1]*	*3.4 [1.2, 5.0]*	*6.12 (0.85, 44.00)*	*0.072*	**4.9 [2.8, 7.1]**	**3.3 [2.0, 4.1]**	**3.20 (1.40, 7.34)**	**0.006**
Ang 2:1 ratio	**0.48 [0.25, 0.87]**	**0.21 [0.10, 0.31]**	**2.29 (1.16, 4.46)**	**0.016**	**0.28 [0.20, 0.46]**	**0.20 [0.01, 0.31]**	**5.72 (1.07, 30.50)**	**0.041**	**0.28 [0.20, 0.46]**	**0.21 [0.01, 0.31]**	**2.56 (1.35, 4.87)**	**0.004**
sICAM (ng/mL)	**276.0 [160.9, 484.7]**	**197.2 [150.1, 346.8]**	**14.11 (1.4, 137.83)**	**0.023**	*265.1 [146.3, 398.8]*	*161.0 [125.0, 222.9]*	*26.72 (0.84, 846.9)*	*0.062*	**262.0 [146.3, 409.1]**	**179.9 [144.0, 272.6]**	**10.25 (2.04, 51.34)**	**0.005**
sVCAM (ng/mL)	1236.6 [950.8, 1544.3]	1154.5 [884.6, 1071.0]	2.38 (0.56, 10.04)	0.239	1166.6 [795.2, 1410.3]	1149.9 [861.1, 1552.7]	1.66 (0.26, 10.67)	0.594	1289.7 [950.8, 1541.1]	1168.0 [886.2, 1647.3]	2.77 (0.80, 9.56)	0.149

*aOR* adjusted odds ratio, *CI* confidence interval, *Ang-1* angiopoietin-1, *Ang-2* angiopoietin-2, *Ang2:1* ratio of Ang-2 to Ang-1, *sICAM* soluble intercellular adhesion molecule-1, *sVCAM* soluble vascular adhesion molecule 1, *IQR* inter-quartile range

*P-value for conditional logistic regression for sex, lethargy and prior antibiotic exposure for Death outcome; sex and prior antibiotic exposure for Bacteremia outcome; sex, lethargy, temperature and prior antibiotic exposure for combined outcome. Biomarkers were log transformed

Comparisons with p-value less than or equal to 0.05 marked in bold, and less than or equal to 0.10 marked in italics

**Table 3:** Angiogenic biomarkers tertiles and relative odds of death

Biomarker	N	Tertile 1 aOR	Tertile 2 aOR (95% CI)	Tertile 3 aOR (95% CI)	p-value for trend
Ang-1	70	1.00 (ref)	0.37 (0.09, 1.52)	0.39 (0.09, 1.62)	0.301
*Ang-2*	*70*	*1.00 (ref)*	*1.39 (0.26, 7.36)*	*5.27 (1.15, 24.07)*	*0.061*
**Ang 2:1 ratio**	**70**	**1.00 (ref)**	**0.57 (0.09, 3.54)**	**4.82 (1.22, 19.03)**	**0.013**
sICAM	70	1.00 (ref)	0.50 (0.09, 2.75)	2.43 (0.60, 9.90)	0.116
sVCAM	70	1.00 (ref)	0.95 (0.22, 4.11)	1.40 (0.36, 5.45)	0.834

*aOR* adjusted odds ratio, *CI* confidence interval, *Ang-1* angiopoietin-1, *Ang-2* angiopoietin-2, *Ang2:1* ratio of Ang-2 to Ang-1, *sICAM* soluble intercellular adhesion molecule-1, *sVCAM* soluble vascular adhesion molecule-1. P-value for trend using conditional logistic regression adjusted for sex, lethargy and prior antibiotic exposure

Comparisons with *p*-value less than or equal to 0.05 marked in bold and 0.05–0.10 marked in italics

Because Ang-1 and Ang-2 can have competitive effects that contribute to microvascular permeability, the ratio of the circulating levels of these two ligands was examined. The median Ang-2:1 ratio was 0.48 [IQR: 0.25, 0.87] among infants who died compared to 0.21 [IQR: 0.10, 0.31] among survivors (aOR 2.29, *p* = 0.016) (Fig. 2 and Table 2). Young infants whose Ang-2:Ang-1 ratio at admission was in the top tertile had 4.82-fold increased odds of death compared with infants in the bottom tertile (p-trend = 0.013) (Table 3).

Higher circulating Ang-2 levels are often associated with increased levels of sICAM-1 and sVCAM-1, reflective of endothelial activation. We examined the association between these downstream targets of Ang-2 signaling and infant sepsis outcomes. Median circulating sICAM-1 concentrations at admission were higher among the septic infants who subsequently died compared with those who survived (276.0 ng/mL [IQR: 160.9, 484.7] vs 197.2 ng/mL [IQR: 150.1, 346.8], aOR 14.11, *p* = 0.023) (Fig. 2, Table 2). When sICAM-1 was categorized into tertiles, the odds of death increased between the first and last tertiles but the trend was not statistically significant (*p* = 0.116) (Table 3). Soluble VCAM-1 concentrations were not associated with the mortality outcome (Fig. 2, Tables 2 and 3).

### The Ang-2:Ang-1 ratio is associated with bacteremia

Among the study cohort, 10 infants had microbiologically confirmed bacterial bloodstream infections, of which three died. Ang-2 concentrations trended higher in bacteremic infants than in non-bacteraemic infants (median 4.1 ng/mL vs 3.4 ng/mL, aOR 6.12, *p* = 0.072). When combined with Ang-1, the ratio of Ang-2:Ang-1 was significantly associated with bacteremia (aOR 5.72, *p* = 0.041) (Fig. 2, Table 2). Soluble-ICAM-1 concentrations trended towards higher concentrations among bacteremic infants than their controls (265.1 ng/mL vs 161.0 ng/mL, aOR 26.72, *p* = 0.062), but the small sample size limits analytical power. There was no difference in Ang-1 or sVCAM-1 concentrations among bacteremic infants compared with their controls.

### Increased circulating markers of endothelial activation at admission are associated with clinical outcomes among young infants

When the primary and secondary outcome groups were combined, the plasma Ang-2 concentration was significantly associated with the Combined Outcome group (aOR 3.20, *p* = 0.006), as was the Ang-2:Ang-1 ratio (aOR 2.56, *p* = 0.004). Soluble ICAM-1 was also significantly higher among these cases compared with the controls (262.0 ng/mL [IQR: 146.3, 409.1] vs 179.9 ng/mL [IQR: 144.0, 272.6], aOR 10.25, *p* = 0.005). Plasma Ang-1 levels displayed a trend towards lower values among the Combined Outcome group compared to the Controls group (11.2 ng/mL [IQR: 5.7, 20.4] vs 15.2 ng/mL [IQR: 8.9, 25.0], aOR 0.54, *p* = 0.089). Soluble-VCAM-1 was not associated with the Combined Outcome (Table 2).

## Discussion

Sepsis remains a significant cause of global infant and child mortality [1]. Early administration of antimicrobial therapy and supportive care can improve outcomes, however early recognition is challenging due to the subtle presentation of sepsis in the newborn. Despite numerous clinical scoring tools to help identify infants at risk for septicemia and death, clinical evaluation of sepsis severity and prognostication remains imprecise [48, 50] and ancillary laboratory investigations are costly and frequently not available in the settings where most neonatal deaths occur. In agreement with previous studies, we found the prognostic utility of typical clinical indicators of infection such as temperature, heart rate, respiratory rate, and lethargy, to be limited with no significant differences observed between infants who died of sepsis compared to those who survived (Table 1). In cases of adult sepsis, determining the levels of immune and endothelial dysfunction at clinical presentation appears to have utility in triage and prognostication but this has not been well studied in the context of neonatal sepsis.

In this study we tested the hypothesis that circulating markers of endothelial dysfunction would identify young infants with life-threatening infections when they first present to a health care facility. We showed an association between biomarkers of endothelial activation and mortality among young infants aged ≤59 days with suspected sepsis at presentation to a pediatric referral centre in a resource-poor setting. Young infants who subsequently died of sepsis had increased circulating concentrations of Ang-2 and the Ang-2:1 ratio at presentation compared with age- and birthweight-matched infants who survived. Plasma levels of sICAM-1, a downstream target of the Angiopoietin-Tie2 pathway, was also significantly associated with mortality. A similar trend was observed among infants with culture-proven bacteremia, but the low rate of bacteremia in this cohort (due in part to pre-hospital antibiotic use) limited analytic power. An increased ratio of circulating Ang-2:Ang-1 was significantly associated with bacteremia, and both Ang-2 and sICAM-1 displayed positive trends with this outcome despite the small number of cases.

### Circulating Angiopoietins are associated with clinical outcomes of sepsis in young infants

This study adds to a growing body of evidence implicating microvascular endothelial injury in the pathophysiology of severe sepsis. Widespread activation of the endothelium triggers endothelial cell dissociation and microvascular leak resulting in hemodynamic collapse and the multi-organ failure associated with septic shock [9, 10, 13]. The Angiopoietin-Tie2 axis is emerging as a critical regulator of the microvascular response to infection [29] and may provide novel targets for intervention to improve outcomes [51, 52].

Studies of sepsis from North American academic hospitals have profiled protein markers of endothelial activation in both the adult and pediatric populations. Among adults, circulating levels of angiopoietins obtained on transfer to the ICU have shown increased circulating concentrations of Ang-2 and decreased levels of Ang-1 in patients who succumbed to sepsis [7, 53]. Mikacenic et al. also found that these endothelial biomarkers correlated with sepsis severity independently of the degree of inflammatory response. Similarly, among adults initially presenting to the Emergency Department with sepsis, Ang-2 levels correlated with sepsis severity and death [17]. Studies involving both young (10 months to 32 months) and older (9 years to 13 years) children with sepsis found that circulating concentrations of Ang-2 at the time of transfer to the Pediatric ICU correlated with sepsis severity and death [44, 45]. Outside of North America, a study conducted in Blantyre, Malawi involving 293 septic children aged two months to 16 years again found mortality to be associated with increased levels of Ang-2 and decreased levels of Ang-1 in plasma samples collected on admission to hospital [46].

This study provides new evidence for a role of the Angiopoietin-Tie2 axis in septic infants less than two months of age. Similar to patterns observed in adults and older children, the circulating concentrations of angiopoietins were associated with clinically significant end-points of sepsis. Increased levels of Ang-2 were associated with mortality and trended with bacteremia outcomes, but our study was not powered for the latter outcome. Ang-1 levels did not vary with mortality or bacteremia outcomes, consistent with previous pediatric studies [44]. Overall, these findings suggest that despite the immaturity of the vascular endothelium in neonates and young infants, the initiating mechanisms of sepsis and vascular leak are regulated by similar pathways as those now well-characterized in older populations. The angiopoietin-Tie2 pathway may therefore serve as both a clinically important diagnostic marker and a therapeutic target for future interventions aimed to mitigate the effects of sepsis and microvascular leak in this vulnerable neonatal population [52].

### Soluble-ICAM-1 is associated with clinical outcomes of sepsis in young infants

The activation of the vascular endothelium by Ang-2 results in an increase in endothelial surface expression of endothelial-leukocyte adhesion molecules including ICAM-1 and VCAM-1 [9]. After the initiation of leukocyte rolling along an activated endothelium, ICAM-1 and VCAM-1 facilitate the firm adhesion of leukocytes with endothelial cells allowing for transendothelial migration and inflammation of surrounding tissues that may ultimately contribute to end organ damage [54–57]. The shedding of sICAM-1 and sVCAM-1 from the endothelial surface is brought about by the activity of proteolytic proteins called sheddases and may represent the initial downregulation phase of the sepsis response [58]. Levels of these circulating adhesion molecules may be indicative of the degree of endothelial activation as well as a host mechanism to limit the sepsis response by binding leukocytes in circulation and preventing their adherence and diapedesis across the endothelial barrier. The functional significance of the soluble adhesion proteins in newborn sepsis requires further study within larger cohorts.

Several studies among adults have established positive associations between circulating levels of sICAM-1 and SIRS, sepsis and sepsis severity including hemodynamic shock, multi-organ failure, and death [32–36, 54, 59–61]; findings from the neonatal and newborn populations have been less consistent. Berner et al. and Dollner et al. did not find an association between sICAM among septic infants [37, 38]. In contrast, studies by Hansen et al., Apostolu et al., and Figueras et al. found increased levels of sICAM-1 among septic infants compared with controls [40, 43, 62], and Edgar et al. demonstrated that sICAM-1 levels predicted infection in newborns [41, 42]. Interestingly even in non-septic newborns, levels of circulating sICAM-1 have been shown to increase over the first week of life; by 30 days of life, plasma concentrations can exceed those of healthy adults [63]. In our study, circulating sICAM-1 concentration in young infants on presentation to a pediatric hospital was positively associated with mortality and culture-proven bacteremia after matching on age. Within this small subset of bacteremic infants (*n* = 10, ages all less than 30 days), those who succumbed to sepsis (*n* = 3) had higher circulating levels of sICAM-1 than those who survived (*n* = 7) (median values 398.0 ng/mL vs 182.5 ng/mL, respectively).

Among adults, the association between sVCAM-1 and sepsis severity has been variable with some studies demonstrating a positive association between sVCAM-1 and sepsis outcomes [34, 53], while others showed no association with sepsis severity or mortality [36]. Soluble-VCAM-1 levels have not been as extensively studied in newborns. One reported study showed that sVCAM-1 levels only increased among septic infants with culture-proven bacteremia, as opposed to the septic, culture-negative comparators [43]. In our study, sVCAM-1 levels were not significantly associated with either mortality or bacteremia.

The discrepant associations between soluble adhesion molecules and sepsis, and the heterogeneity between the adult and newborn populations, suggests potential developmental changes in the pathophysiology of sepsis. In a review by Zonneveld et al., circulating endothelial adhesion molecule concentrations among healthy and septic individuals were studied across neonatal, child and adult age groups. Overall, the concentrations of soluble adhesion molecules, including sICAM-1 and sVCAM-1, increased during sepsis but both the relative and absolute extent of increase were markedly lower among neonates compared with the older age groups. The authors posit that the failure to robustly upregulate circulating sICAM-1 may be reflective of either an immature, hyporesponsive immune response, or age-related differences in the production of sheddases [58]. The functional significance of the soluble adhesion proteins in newborn sepsis requires further study.

### Study limitations

This study was conducted in a resource-poor setting where tools for clinical assessment are limited and laboratory indicators of sepsis severity were not available. Medical management of the septic newborns was left to the discretion of the treating physician and this is a potential source of bias. Notably, among the parent-study cohort of septic infants from which our study sample was derived, the rate of bacteremia was 3.1%, less than expected; moreover, the pathogens typically isolated from newborns in Bangladesh, including *Staphylococcus aureus* and gram negative organisms [64, 65], were not predominant in this cohort (Additional file 1: Table S1). Importantly, 44% of our study cohort had antibiotic exposure prior to blood sampling for culture and 45% of those with negative blood cultures received antibiotics prior to sampling. Thus the rate of bacteremia identified by blood culture may underestimate the true prevalence of bacteremia in this cohort, and the reported organisms may not have identified all of the causative pathogens. Finally, due to changes in specimen storage in Bangladesh, samples collected from the first 71 enrolled infants were not available for analysis. Additional prospective studies will be required to validate the findings of this study.

## Conclusions

Sepsis and its sequelae remain leading causes of infant morbidity and mortality, particularly in resource-poor settings where rates are highest, and the availability of intensive supportive care are the lowest. Our results demonstrate that circulating markers of endothelial dysfunction at the time that infants present for medical attention have the potential to risk stratify those in most need of aggressive medical support. If these findings are externally validated by additional prospective studies, point-of-care tests incorporating these markers may enable rapid triage of critically ill neonates at risk of death from sepsis.

## Additional file

Additional file 1: **Table S1.** Blood culture isolates from bacteremic infants. Bacterial isolates from blood cultures obtained by venipuncture on admission of septic young infants age < 59 days to a pediatric facility in Sylhet, Bangladesh. (DOCX 16 kb)

## Abbreviations

Ang-1: Angiopoietin-1
Ang-2: Angiopoietin-2
aOR: adjusted odds ratio
ELISA: Enzyme-Linked Immunosorbent Assay
ICAM-1: Intercellular adhesion molecule-1
NFkB: Nuclear Factor kappa-light-chain-enhancer of activated B cells
sICAM: Soluble Intercellular adhesion molecule-1
SIRS: Systemic inflammatory response syndrome
SOMCH: Sylhet MAG Osmani Medical College Hospital
sVCAM: Soluble Vascular cell adhesion molecule-1
VCAM-1: Vascular cell adhesion molecule-1
WHO: World Health Organization
