# Supplementary material for: Biomarkers of endothelial dysfunction predict sepsis mortality in young infants: a matched case-control study
Source: BMC Pediatr. 2018 Mar 23;18:118. doi: 10.1186/s12887-018-1087-x (PMC5866512; doi:10.1186/s12887-018-1087-x)
Supplement: Supplementary file 1 — Table S1. Blood culture isolates from bacteremic infants. Bacterial isolates from blood cultures obtained by venipuncture on admission of septic young infants age < 59 days to a pediatric facility in Sylhet, Bangladesh. (DOCX 16 kb) [file 12887_2018_1087_MOESM1_ESM.docx]

| Types/Names of isolates | **Total** | **Death** | **Bacteremia,  no death** |
| --- | --- | --- | --- |
| Gram Positive Isolates | 7 | 2 | 5 |
| *Staphylococcus aureus* | 4 | 0 | 4 |
| *Streptococcus pyogenes* | 1 | 1 | 0 |
| *Streptococcus pneumoniae* | 1 | 1 | 0 |
| alpha-hemolytic Streptococcus | 1 | 0 | 1 |
| Gram Negative Isolates | 3 | 2 | 1 |
| *Escherichia coli* | 1 | 1 | 0 |
| *Enterobacter sp.* | 1 | 1 | 0 |
| *Acinetobacter sp.* | 1 | 0 | 1 |
| All Positive Blood Cultures | 10 | 4 | 6 |

**Additional file 1 Table S1**

Blood culture isolates from bacteremic infants. Bacterial isolates from blood cultures obtained by venipuncture on admission of septic young infants age <59 days to a pediatric facility in Sylhet, Bangladesh.
